# Invasive Fusariosis Among Patients with Acute Leukemia: A 12-Year Single-Center Experience in a Middle-Income Country

**DOI:** 10.1007/s11046-026-01068-3

**Published:** 2026-03-08

**Authors:** Raquel Keiko de Luca Ito, Patricia Rodrigues Bonazzi Pontes, Adriana Satie Goncalves Kono Magri, Karim Yaqub Ibrahim, Odeli Nicole Encinas Sejas, Jessica Toshie Katayose Goto, Juliana Pereira, Marcia de Souza Carvalho Melhem, Marcello Mihailenko Chaves Magri, Edson Abdala

**Affiliations:** 1 São Paulo State Cancer Institute (ICESP), University of São Paulo School of Medicine, São Paulo, Brazil; 2 School of Medicine, Federal University of Mato Grosso Do Sul, Campo Grande, Brazil; 3 Central Laboratory Division, Hospital das Clínicas, University of São Paulo School of Medicine, São Paulo, Brazil; 4 Department of Infectious and Tropical Diseases, University of São Paulo School of Medicine, São Paulo, Brazil; 5 Medical Investigation Laboratory (LIM/53), Hospital das Clínicas, University of São Paulo School of Medicine, São Paulo, Brazil

**Keywords:** Invasive fusariosis, Acute leukemia, Invasive fungal disease, Neutropenia

## Abstract

**Introduction:**

Invasive fusariosis (IF) is one of the most aggressive mold infections in patients with acute leukemia, characterized by rapid dissemination, high rates of fungemia, and limited antifungal susceptibility. Its impact is particularly severe in middle-income countries, yet single-center data from these settings remain scarce.

**Materials and Methods:**

We conducted a retrospective study of all patients with acute leukemia and proven IF, diagnosed according to EORTC/MSG criteria, from January 2011 to August 2023. Demographic, clinical, therapeutic, and outcome variables were analyzed.

**Results:**

Twenty-six patients were identified. Median age was 46.5 years, and 53.8% were female. Acute myeloid leukemia was the most frequent underlying condition. All patients presented with severe neutropenia at diagnosis. Disseminated disease occurred in 84.6% of cases; two patients had isolated fungemia and two had localized skin disease. Cutaneous lesions were present in 73% of patients, and 31% had pulmonary involvement. Antifungal prophylaxis, mainly fluconazole or anidulafungin, was used in 69.2% of cases. Treatment consisted of lipid formulations of amphotericin B, either alone or combined with voriconazole. Thirty-day mortality reached 38.5%. Mortality was significantly higher among patients undergoing re-induction therapy, whereas those who had received antifungal prophylaxis exhibited lower mortality.

**Discussion:**

IF in acute leukemia was associated with extensive dissemination and substantial early mortality. Mortality rates were higher in patients in re-induction therapy and lower in those who received antifungal prophylaxis. The role of combination antifungal therapy requires further investigation.

## Introduction

Invasive fusariosis (IF) represents one of the most challenging invasive mold infections encountered in patients with prolonged neutropenia, combining rapid clinical progression with intrinsically limited therapeutic options. Reflecting this clinical threat, the World Health Organization recently classified IF as a fungal infection of major global priority, driven by its expanding epidemiological footprint, limited therapeutic arsenal, and consistently high mortality in vulnerable populations [1].

*Fusarium* is a ubiquitous mold, found in soil and water in the environment. It is usually considered a plant pathogen, but has been increasingly associated with opportunistic infections of humans and other animals [2, 3]. *F. solani* species complex and *F. oxysporum* species complex are responsible for the majority of infections [4], and can cause a wide spectrum of clinical manifestations, including localized superficial or invasive infection and disseminated disease, depending on the immune status of the host and the portal of entry [2, 5, 6]. In immunocompromised patients, airways are the main portal of entry, followed by skin at the site of tissue breakdown and possibly the mucosal membranes [6]. IF is currently considered the first or the second most frequent mold infection in immunocompromised patients in several series [7–11]. With the adoption of antifungal prophylaxis and the introduction of new extended-spectrum azole antifungal agents, used for prevention and treatment of invasive aspergillosis, some mold infections so far considered rare, such as IF, have emerged.

In patients with acute leukemia, particularly those exposed to intensive chemotherapy and prolonged neutropenia, the infection often progresses explosively, with skin, lungs, and sinuses serving as early indicators of disseminated disease. Despite this well-recognized severity, contemporary data from middle-income countries remain remarkably limited. This knowledge gap constrains clinical decision-making precisely in settings where the burden of hematologic malignancies is substantial.

## Materials and Methods

This retrospective study included patients with acute leukemia and proven IF, defined according to the modified European Organization for Research and Treatment of Cancer/Mycoses Study Group (EORTC/MSG) criteria [12], diagnosed between January 2011 and August 2023 at the São Paulo State Cancer Institute (*Instituto do Câncer do Estado de São Paulo—*ICESP), Brazil. *Fusarium* spp. isolates were identified by proteomic analysis using MALDI-TOF MS (VITEK, bioMérieux, USA) at the Central Laboratory Division (*Divisão de Laboratório Central*) of *Hospital das Clínicas*, University of São Paulo School of Medicine, Brazil. For species-level identification, genomic DNA extraction followed by amplification and sequencing of the ITS region and the TEF1α gene was performed, as these markers provide high discriminatory power for resolving *Fusarium* species [13–15]. The traditional nomenclature of *Fusarium* spp. was used according to the proposed global consensus guideline for fungal name changes by de Hoog et al., [16, 17].

Demographic, clinical, and laboratory data from patients were obtained: age, sex, underlying hematological disease, presence of comorbidities, state of disease (active, remission, relapse/refractory disease), stage of treatment (induction, consolidation or re-induction), clinical presentation (localized versus disseminated infection or fungemia alone), days of neutropenia, antifungal therapy and previous use of antifungal prophylaxis (fluconazole or anidulafungin). Patients with the concurrent involvement of 2 or more noncontiguous sites were defined as disseminated infection. Cases of fungemia were not defined as disseminated infection unless another organ was involved. The involvement of only one organ (e.g., skin, sinuses, etc.) in the absence of fungemia was defined as localized infection [16].

Neutropenia was defined as an absolute neutrophil count < 500 cells/mcL, or an absolute neutrophil count < 1000 cells/mcL with an expected decline to 500 cells/mcL over the next 48 h. Severe neutropenia was defined as an absolute neutrophil count < 100 cells/mcL. Prolonged neutropenia was defined as an absolute neutrophil count below the threshold for more than seven consecutive days [18].

Mortality up to 30 days after the diagnosis of IF was the outcome evaluated. Thirty-day mortality was described according to each evaluated qualitative characteristic, and its association was tested using Fisher’s exact test or likelihood ratio tests, and to each quantitative characteristic using Student's t-test or Mann–Whitney tests [19]. Unadjusted odds ratios (OR) with 95% confidence intervals were estimated for each evaluated characteristic using bivariate logistic regression [20]. For the analysis, IBM-SPSS for Windows version 22.0 was used. The tests were conducted with a significance level of 5%.

## Results

**Fig. 1:**
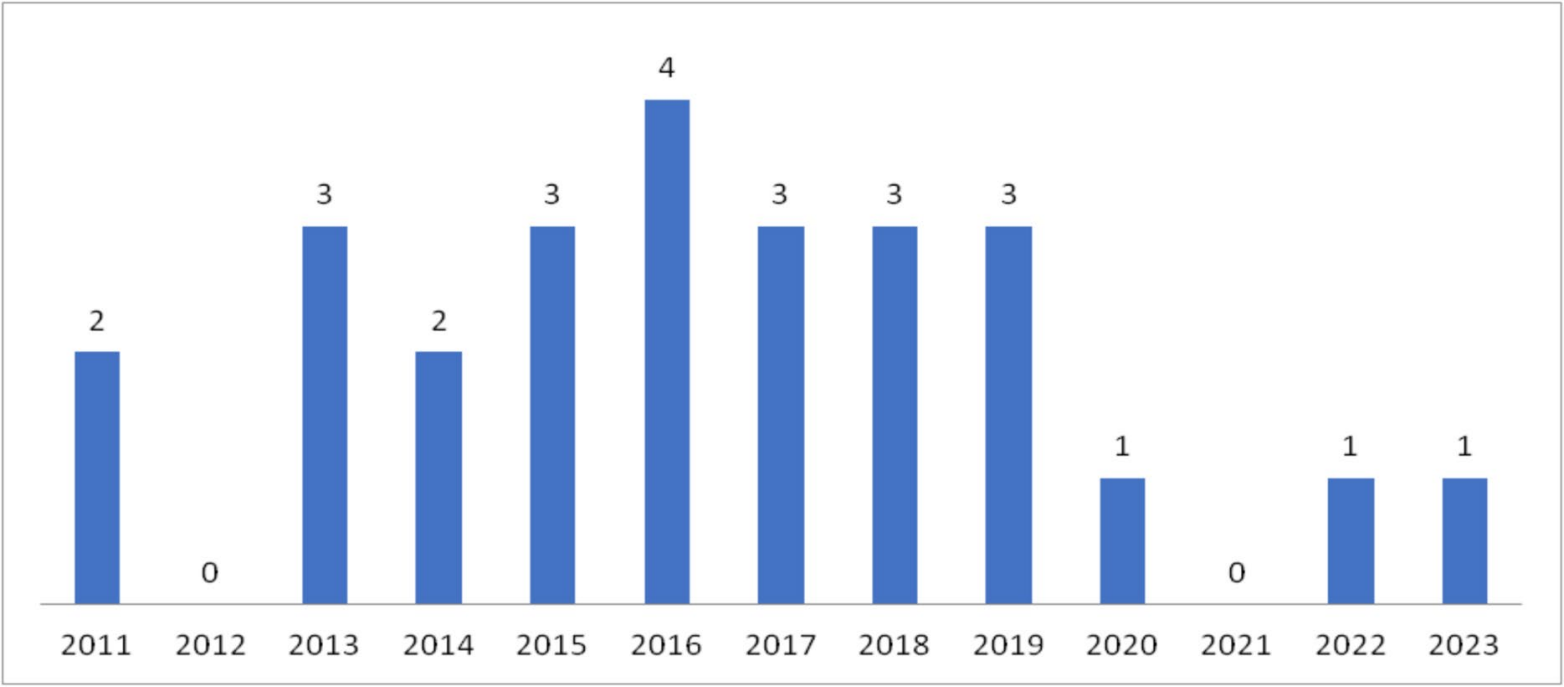
Cases of *Fusarium* infections in patients with acute leukemia per year, from January 2011 to August 2023

Twenty-six patients with acute leukemia and IF were identified. The annual distribution of cases is shown in Fig. 1. The 30-day mortality rate was 38.5% (10 deaths). Demographic and clinical characteristics, as well as comparisons between survivors and non-survivors within 30 days of diagnosis, are presented in Tables 1 and 2, respectively.

**Table 1:** Characteristics of 26 patients with acute leukemia and invasive fusariosis

Characteristic	(N = 26)
*Age (yr)*	
Mean ± SD	45.4 ± 15.9
Median (25th; 75th percentiles)	46.5 (31; 61.3)
*Sex, n (%)*	
Female/male	14 (53.8) / 12 (46.2)
*Hematological disease, n (%)*	
Acute myeloid leukemia (AML)	16 (61.5)
Acute lymphocytic leukemia (ALL)	7 (26.9)
Acute undifferentiated leukemia	3 (11.5)
*Comorbidities, n (%)*	
Hypertension	6 (23.1)
Diabetes	2 (7.7)
Smoking	6 (23.1)
Other oncological disease	3 (11.5)
*Stage of disease, n (%)*	
Active	18 (69.2)
Relapse/refractory	8 (30.8)
*Stage of treatment, n (%)*	
Induction	20 (76.9)
Maintenance	1 (3.8)
Re-induction	5 (19.2)
*Days of neutropenia (N* < *500)*	
Mean ± SD	22.2 ± 11.9
Median (25th;75th percentiles)	20 (12.8; 33.3)
*Days of severe neutropenia (N* < *100)*	
Mean ± SD	17.6 ± 10.2
Median (25th;75th percentiles)	14.5 (11.8; 20.5)
*Species complex*	
*F. solani*	14 (53.8)
*Fusarium* spp.	12 (46.2)
*Clinical presentation, n (%)*	
Fungemia	2 (7.7)
Localized (skin)	2 (7.7)
Disseminated	22 (84.6)
*Antifungal therapy, n (%)*	
Amphotericin B (AmB)	13 (50)
AmB + Voriconazole (VCZ)	13 (50)
Antifungal prophylaxis, n (%)	18 (69.2)
Death within 30 days, n (%)	10 (38.5)

**Table 2:** Comparison between survivors and non-survivors 30 days after diagnosis of fusariosis in patients with acute leukemia

Variable	Death within 30 days		OR	CI (95%)		*p*
	No (*N* = 16)	Yes (*N* = 10)		Lower	Upper	
*Age (yr)*			1.01	0.96	1.07	0.612**
Mean ± SD	44.1 ± 15.6	47.4 ± 16.9				
Median (25th;75th percentiles)	46.5 (31.5; 59)	49 (30.8; 64.3)				
*Sex, n (%)*						0.248
Female	7 (50)	7 (50)	1.00			
Male	9 (75)	3 (25)	0.33	0.06	1.78	
*Hematological disease, n (%)*						0.195#
AML	12 (75)	4 (25)	1.00			
ALL	3 (42.9)	4 (57.1)	4.00	0.61	26.12	
Acute undifferentiated leukemia	1 (33.3)	2 (66.7)	6.00	0.42	85.25	
*Comorbidities, n (%)*						> 0.999
No	7 (63.6)	4 (36.4)	1.00			
Yes	9 (60)	6 (40)	1.17	0.23	5.81	
*Hypertension, n (%)*						0.163
No	14 (70)	6 (30)	1.00			
Yes	2 (33.3)	4 (66.7)	4.67	0.67	32.74	
*Diabetes, n (%)*						0.138
No	16 (66.7)	8 (33.3)	1.00			
Yes	0 (0)	2 (100)	&			
*Smoking, n (%)*						0.644
No	13 (65)	7 (35)	1.00			
Yes	3 (50)	3 (50)	1.86	0.29	11.76	
*Other cancer, n (%)*						> 0.999
No	14 (60.9)	9 (39.1)	1.00			
Yes	2 (66.7)	1 (33.3)	0.78	0.06	9.89	
*Stage of disease, n (%)*						0.189
Active	13 (72.2)	5 (27.8)	1.00			
Relapse/refractory	3 (37.5)	5 (62.5)	4.33	0.74	25.29	
*Stage of treatment, n (%)*						**0.028#**
Induction	15 (75)	5 (25)	1.00			
Maintenance	0 (0)	1 (100)	&			
Re-induction	1 (20)	4 (80)	12.00	1.07	134.11	
*Days of neutropenia* *(N* < *500)*			1.05	0.97	1.12	0.363£
mean ± SD	19.9 ± 10.4	25.8 ± 13.7				
median (25th;75th percentiles)	17 (13.3; 24)	27.5 (11.8; 34.3)				
*Days of severe neutropenia (N* < *100)*			1.04	0.96	1.13	0.517£
Mean ± SD	16 ± 9.2	20.2 ± 11.8				
Median (25th;75th percentiles)	14 (12; 18.8)	17.5 (8.8; 33.3)				
*Clinical presentation, n (%)*						0.349#
Fungemia	1 (50)	1 (50)	1.00			
Localized (skin)	2 (100)	0 (0)	&			
Disseminated	13 (59.1)	9 (40.9)	0.69	0.04	12.57	
*Antifungal therapy, n (%)*						0.226
AmB	6 (46.2)	7 (53.8)	1.00			
AmB + VCZ	10 (76.9)	3 (23.1)	0.26	0.05	1.39	
*Antifungal prophylaxis, n (%)*						**0.026**
No	2 (25)	6 (75)	1.00			
Yes	14 (77.8)	4 (22.2)	0.10	0.01	0.67	

Fisher's exact test; # Likelihood Ratio test; **Student’s t-test (unpaired t-test); £ Mann–Whitney test; & Unable to estimate. *P* value < 0.05 was considered statistically significant

The median age was 46.5 years, and slightly more than half were female. Acute myeloid leukemia predominated (61.5%). Most patients had active disease at the time of infection and were receiving first-induction chemotherapy. Comorbidities were common, particularly hypertension and smoking, each present in 23.1% of cases. All patients were neutropenic, with a median duration of neutropenia of 20 days and severe neutropenia of 14.5 days. Species-level identification was performed in 14 of 26 isolates, all of which were identified as *F. solani* species complex (FSSC). Disseminated fusariosis was the leading clinical presentation (84.6%). Antifungal treatment was evenly divided between lipid amphotericin B (AmB) monotherapy and combination therapy with AmB plus voriconazole (VCZ). Notably, 69.2% of patients had received prior antifungal prophylaxis.

Among the 26 patients evaluated, 10 died within 30 days of diagnosis. The mean age was slightly higher among non-survivors (47.4 ± 16.9 vs. 44.1 ± 15.6 years). Deaths occurred in 25% of patients with acute myeloid leukemia, 57.1% in those with acute lymphocytic leukemia, and 66.7% in those with undifferentiated leukemia. Regarding comorbidities, deaths occurred in 40% of those with some comorbidity, and in 36.4% of those with none. In patients with relapsed or refractory disease we observed 62.5% of deaths, in contrast to 27.8% with active disease. The median duration of neutropenia was higher among non-survivors (27.5 vs 17 days). The progression to death occurred in 53.8% of patients receiving AmB monotherapy, and in 23.1% of those receiving combination therapy.

Individually, patients in the re-induction treatment phase had a statistically higher frequency of mortality up to 30 days (80% vs 25%, *p* = 0.028). Patients who received antifungal prophylaxis had a statistically lower frequency of mortality up to 30 days (22.2% vs 75%, *p* = 0.026). Since the sample size was very small and the characteristics that were statistically significant in the bivariate analyses have intersections with zero cases, it was not possible to estimate a multivariable model.

## Discussion

IF remains a severe and highly morbid infection in patients with acute leukemia, particularly in the context of prolonged neutropenia. In this cohort, most patients had acute myeloid leukemia and were receiving first-induction chemotherapy at the time of infection, and disseminated disease was the predominant presentation. All individuals exhibited severe neutropenia, and skin and pulmonary involvement were frequent findings. The 30-day mortality rate of 38.5% reflects the seriousness of IF in this setting, and differences between survivors and non-survivors were mainly observed according to treatment phase and prior antifungal prophylaxis.

The largest case series of *Fusarium* spp. infections are multicenter studies in patients with hematopoietic cell transplantation (HCT) and hematological malignancies [16, 21–23], with a limited number of cases from each site. In patients with fusariosis and hematological malignancies, acute leukemia was the most frequent underlying condition [24–26]. Severe and prolonged neutropenia have been associated with the development of invasive and disseminated disease, with higher mortality rates, compared to localized disease [16, 27]. IF has also been reported in patients with other underlying conditions, such as solid tumors, diabetes, chronic cardiac or lung disease, rheumatoid arthritis, solid organ transplantation and burn patients [16, 26, 28]. In this study, we described a series of 26 cases of IF in patients with acute leukemia. To the best of our knowledge, this is one of the largest single-center series in patients in this scenario. The presence of acute myeloid leukemia, severe and prolonged neutropenia, and use of immunosuppressive therapies were the conditions most frequently associated with fusariosis in our patients, as has been described in other series [16, 21, 26].

Regarding clinical presentation, most study patients had disseminated disease. This is the most common clinical presentation of IF in immunocompromised patients, especially those with hematological malignancies, as well as in our case series [6, 25]. In most cases, the patients were febrile and developed disseminated and characteristic skin lesions, with the isolation of mold in blood cultures, as in other series [6, 8, 16, 21]. Fungemia, characterized by the persistence of fever despite broad-spectrum antimicrobial therapy and positive blood cultures, is also a frequent clinical presentation in neutropenic patients and can occur occasionally among non-neutropenic patients with a central vascular catheter [6]. Skin-localized infection is more frequent in immunocompetent patients and is usually associated with the portal of entry of the microorganism [6]. We identified two patients with fungemia alone and two cases with localized cutaneous disease.

Skin lesions can be the primary site of infection or may be associated with the development of disseminated disease, preceding the isolation of fungi in blood cultures [29]. The typical lesion is a red or gray macule or papule, with a central eschar or ulceration [30]. Skin lesions were present in 17/22 patients with disseminated disease (77.3%), but a cutaneous portal of entry (*tinea pedis* and onychomycosis) was found only in 2 cases.

Pulmonary disease is another clinical presentation found in patients with hematological malignancies, characterized by the presence of respiratory symptoms and bilateral nodules or cavitations in the lungs [6]. Out of 22 patients with disseminated disease, 8 (36.4%) had lung involvement. In immunocompromised patients, the sinuses may also be involved, as part of disseminated disease [6]. Out of 8 patients with disseminated disease and fungal sinusitis, *Fusarium* was isolated from nasal mucosa biopsy in 3 cases.

Infections associated with invasive devices, such as bloodstream infections related to central vascular catheters and peritonitis in patients receiving peritoneal dialysis, are rare [6]. In our case series, the mold was isolated from catheter blood samples in 8 cases (36.4%), but these patients with hematological malignancies had elevated catheter utilization rates. Only one had undergone hemodialysis sessions in the last 72 h preceding fungal isolation.

The optimal treatment strategy for patients with IF has not yet been defined due to the lack of randomized trials [31, 32]. The current global guidelines for the diagnosis and management of rare mold infections strongly recommend VCZ or a lipid formulation of AmB for the primary treatment of IF and recommend against the use of AmB deoxycholate if other active antifungal agents are available [32]. Primary combination therapy with VCZ plus liposomal AmB, with a potential early step-down to monotherapy later is also an approach strongly recommended [32] and was an approach used in many cases at our center due to the severity of the disease and due to the high minimum inhibitory concentration of voriconazole observed in the isolates from our patients [33]. However, correlation between MIC and outcomes was not observed in the literature [23, 34, 35].

The findings of this cohort align with the global patterns described in the WHO systematic review on *Fusarium* spp. infections. In that review, 30-day mortality associated with IF ranged from 42.9 to 66.7% across published studies, values comparable to the 38.5% observed in our setting. The WHO report also highlights substantially higher mortality among neutropenic patients (65.9%) compared with non-neutropenic individuals (28.6%), consistent with our cohort in which all patients presented with prolonged neutropenia. Furthermore, the global systematic review emphasizes the predominance of *Fusarium solani* species complex and the limited susceptibility of isolates to available antifungals, characterized by high MICs for multiple agents, together contributing to the severe outcomes of IF worldwide. Taken together, the results of our study mirror the epidemiological and clinical severity highlighted by the WHO analysis, reinforcing that IF remains a high-mortality infection even in contemporary cohorts.

Although bearing no statistical significance, mortality rates were lower in the patients who received combination therapy. Nonetheless, we could not define whether this mortality reduction was due to the use of voriconazole by itself or due to the antifungal association, once we did not identify any patient who had received monotherapy with voriconazole during the study period. Patients who developed IF in re-induction therapy had significant mortality rates, compared to those in first-induction therapy, but mortality could also be attributed to the severity of the hematological disease. Antifungal prophylaxis was identified as a potential protective factor, although the drugs used had no activity against *Fusarium* spp. Once these patients had a higher risk for invasive fungal diseases, the grade of suspicion for mold infections for them might be higher, and consequently adequate antifungal therapy initiated earlier, compared to those who had not received prophylaxis.

Our study has some limitations. It is a single-center retrospective study, involving only patients with acute leukemia and proven IF; therefore, a selection bias may have occurred because patients with positive cultures are more likely to develop severe and disseminated disease, with higher mortality rates. The therapeutic approach was not uniform and changed over time, impairing the assessment of outcomes. Because of the small size of the sample, it was not possible to perform a multivariate analysis of the characteristics that were statistically significant in the bivariate analyses.

In this cohort of patients with acute leukemia, IF occurred predominantly during periods of profound and prolonged neutropenia and was characterized by a high frequency of disseminated disease. The 30-day mortality rate remained substantial, reflecting the severity of infection in this population. Differences in outcomes were mainly associated with the treatment phase and prior antifungal prophylaxis. Although all patients received lipid formulations of AmB, alone or in combination with VCZ, the optimal therapeutic approach remains uncertain. Larger, multicenter studies are needed to better define prognostic factors and to clarify the impact of combination therapy on outcomes.

## Data Availability

No datasets were generated or analysed during the current study.
